# Autoimmune complications of tyrosine kinase inhibitors in cancer therapy: Clinical insights, mechanisms, and future perspectives

**DOI:** 10.1097/MD.0000000000039928

**Published:** 2024-10-04

**Authors:** Juan Shi, Qingyuan Fu, Quancheng Ma, Zhenzhen Wei, Xiaolian Su, Xiao Li

**Affiliations:** a Department of Pharmacy, The First People’s Hospital of Jinan, Jinan, China; b Department of Neurology, The First People’s Hospital of Jinan, Jinan, China; c Department of Clinical Laboratory, The First People’s Hospital of Jinan, Jinan, China; d Department of Science and Education, The First People’s Hospital of Jinan, Jinan, China; e Department of Obstetrics and Gynecology, The First People’s Hospital of Jinan, Jinan, China; f Department of Clinical Pharmacy, The First Affiliated Hospital of Shandong First Medical University & Shandong Provincial Qianfoshan Hospital, Shandong Engineering and Technology Research Center for Pediatric Drug Development, Shandong Medicine and Health Key Laboratory of Clinical Pharmacy, Jinan, China.

**Keywords:** autoimmune diseases, cancer therapy, immune system dysregulation, preventive strategies, tyrosine kinase inhibitors

## Abstract

The tyrosine kinase inhibitors (TKIs) have emerged as a promising class of novel anticancer drugs, achieving significant success in clinical applications. However, the risk of autoimmune diseases associated with these drugs has raised widespread concerns. In this review, TKI-induced autoimmune diseases are reviewed in order to understand this complex phenomenon through clinical research and molecular mechanism exploration. Despite the relatively low incidence of autoimmune diseases, their potential severity demands heightened attention. The potential mechanisms underlying TKI-induced autoimmune diseases may involve immune system dysregulation, alterations in immune cell function, activation of inflammatory responses, and attacks on self-antigens. Various preventive strategies, including clinical monitoring, personalized treatment, optimization of therapeutic approaches, and patient education and communication, can be employed to effectively address these potential risks. Future research directions should delve into the molecular mechanisms of TKI-induced autoimmune diseases, integrate studies on genetics and immunogenetics, advance the development of novel TKIs, explore the possibilities of combining immunotherapy with TKI treatment, and propel large-scale clinical trials.

## 1. Introduction

In the past decade, tyrosine kinase inhibitors (TKIs) have emerged as a promising class of targeted anticancer drugs, offering new prospects in the treatment of cancer patients.^[1]^ By specifically inhibiting tyrosine kinase signaling pathways, these drugs effectively disrupt the proliferation and survival of tumor cells. However, with the widespread use of these agents, there has been increasing concern about their serious adverse effects.^[2]^Common adverse reactions associated with TKIs include, but are not limited to, gastrointestinal disturbances, cutaneous manifestations, fatigue, kidney injury, and hematologic effects.^[3–5]^ While these adverse effects are known to affect patients’ quality of life, recent research has revealed an even greater concern that TKIs may induce autoimmune diseases.^[6–8]^

Sunder et al proposed a state-of-the-art review, which have systematically reviewed and summarized the adverse reactions associated with TKIs.^[2]^ However, it is important to note that the correlation between TKIs and autoimmune diseases has not received enough attention. Potential issues arising from autoimmune diseases during TKI treatment, such as immune system imbalance and autoimmune organ damage, remain insufficiently elucidated.

Although the incidence of TKI-induced autoimmune diseases is relatively low, once it occurs, it will cause great harm to the patient’s health. The onset of autoimmune diseases may not only lead to organ-specific autoimmune damage but also escalate into systemic autoimmune conditions, presenting formidable challenges to both survival and quality of life.^[9–12]^

This review focuses on the issue of TKI-induced autoimmune diseases, systematically reviewing relevant research, and delving into the potential underlying mechanisms. Our goal is to highlight the need for increased vigilance against this potential adverse effect and to provide a basis for the selection of safer treatment options in clinical practice.

## 2. TKIs and their mechanisms

TKIs, structurally diverse, selectively target specific tyrosine kinase receptors such as epidermal growth factor receptor (EGFR),^[13]^ vascular endothelial growth factor receptor (VEGFR),^[14]^ and platelet-derived growth factor receptor.^[15]^ This specificity allows tailored therapeutic interventions based on the molecular profile of the targeted cancer. For instance, EGFR inhibitors like erlotinib^[16]^ and gefitinib^[17]^ have proven effective in treating non-small cell lung cancer with EGFR mutations, showcasing the precision of TKIs in addressing specific cancer subtypes.

The mechanisms through which TKIs exert their anticancer effects are multifaceted. Primarily, these inhibitors interfere with the phosphorylation of tyrosine residues on target kinases, disrupting downstream signaling cascades. By blocking the activation of pathways crucial for cell proliferation and survival, TKIs induce cell cycle arrest and apoptosis in cancer cells.^[18,19]^ However, this targeted disruption of signaling pathways may also impact the delicate balance of immune regulation, leading to unintended consequences such as the development of autoimmune diseases.

A growing body of emerging evidence suggests that TKIs can disrupt the immune system, potentially leading to autoimmune disorders. The disruption of immune homeostasis may result in the dysregulation of self-tolerance mechanisms, triggering the immune system to attack healthy tissues.^[20,21]^ This phenomenon has been observed in clinical studies, where a subset of patients undergoing TKI treatment has experienced autoimmune organ damage or developed systemic autoimmune diseases.^[7,22–26]^ The use of TKIs requires careful consideration of the delicate balance between their anticancer effects and potential immunomodulatory consequences.

## 3. Association between TKIs and autoimmune diseases

A retrospective study focused on patients undergoing erlotinib treatment for lung cancer revealed that a subset of patients experienced immune-related adverse events during the treatment, such as autoimmune thyroid diseases and skin immune reactions.^[27]^ This finding suggests that TKIs may disrupt immune system balance, leading to the development of autoimmune diseases. Another study, centered around imatinib and its treatment of chronic myeloid leukemia, identified instances where patients undergoing imatinib treatment developed autoimmune diseases, with autoimmune thyroid diseases being particularly pronounced.^[28]^

Upon comprehensive data analysis, it becomes evident that the incidence of TKI-induced autoimmune diseases is relatively low, typically ranging from 0.1% to 26%. Despite this low incidence, once manifested, clinical presentations can be severe, affecting a broad spectrum of organ systems, including but not limited to the thyroid, skin, lungs, and pancreas.^[25]^

In fact, TKIs can trigger a variety of autoimmune diseases, with autoimmune thyroid diseases and rheumatoid arthritis being the most prevalent, as shown in Table 1.^[29–53]^ Clinical practice has shown that patients treated with TKI may experience thyroid dysfunction, manifested by elevated or decreased thyroid hormone levels. In addition, the typical symptoms of rheumatoid arthritis, such as joint pain, swelling, and limited mobility, have been observed in some cases.

**Table 1:** Common types and frequency of autoimmune complications in tyrosine kinase inhibitors.

Tyrosine kinase inhibitors	Molecular target	Autoimmune complications
Afatinib	ErbB1/2/4	ILD 6.33%
Aflibercept	PGF, VEGF-A	GI perforation 1.40% to 5.45%
Alectinib	ALK, RET	ILD 2.64%
Axitinib	VEGFR1/2/3	Thyroid dysfunction 8.2% to 79.70%
Bevacizumab	VEGF-A	ILD 0.89%, colitis 0.48% to 0.84%, GI perforation 0.16% to 9.76%
Binimetinib	MEK1/2	ILD 1.12%, uveitis 1.08%
BRAF nd	–	Peripheral neuropathy 1.61%
Brigatinib	ALK	ILD 6.96%
Buparlisib	PI3Kα/β/γ/δ	GI perforation 1.39%
Cabozantinib	MET, VEGFR1/2/3, ROS1, RET, AXL, NTRK, KIT	Thyroid dysfunction 12.1% to 23.1%
Ceritinib	ALK	ILD 1.07% to 3.25%
Copanlisib	PI3Kα/δ	ILD 7.75%, colitis 0.70%
Crizotinib	ALK, ROS1	ILD 1.81%
Dabrafenib	BRAF, CRAF	Uveitis 0.47%
Dabrafenib + trametinib	BRAF + MEK	Uveitis 0.48% to 0.57%, peripheral neuropathy 3.51%
Dabrafenib + trametinib + pembrolizumab	BRAF + MEK + PD-1	Thyroid dysfunction 13.3%
Dasatinib	BCR-Ab1	Colitis 1.54% to 18.84%
Encorafenib	BRAF	Retinopathy 2.08%
Encorafenib + binimetinib	BRAF + MEK1/2	Retinopathy 19.79%
Erlotinib	EGFR	ILD 2.10% to 5.83%, peripheral neuropathy 9.52%
Gefitinib	EGFR	ILD 0.27% to 6.93%, uveitis 0.45%, peripheral neuropathy 16.18%
Ibrutinib + ofatumumab	BTK + CD20	Peripheral neuropathy 43.66%
Idelalisib	PI3Kδ	ILD 3.16%, colitis 13.95% to 20.00%
Idelalisib + rituximab	PI3K + CD20	ILD 10.00%, 10.91% to 14.06%
Lenvatinib	VEGFR, RET	Thyroid dysfunction 16.4% to 36.5%
Lorlatinib	ALK	ILD 1.76%, peripheral neuropathy 38.89%
Momelotinib	ALK2, JAK1, JAK2	Peripheral neuropathy 10.23% to 47.00%
Motesanib	PDGFR, RET, VEGFR1/2/3, c-Kit	Thyroid dysfunction 5.8% to 40.7%
Nilotinib	BCR-Ab1	Thyroid dysfunction 43.6%
Osimertinib	EGFR	ILD 10.68%
Panitumumab	EGFR	ILD 0.14%
Pazopanib	VEGFR1/2/3	Thyroid dysfunction 12.10% to 15.20%
Ponatinib	BCR-Ab1	Pancreatitis 6.71% to 16.92%
Ruxolitinib	JAK1/2	Peripheral neuropathy 4.61%
Sorafenib	VEGFR1/2/3	Thyroid dysfunction 1.70% to 66.70%, GI perforation 0.69% to 2.03%, pancreatitis 0.34% to 4.35%, uveitis 0.03%, peripheral neuropathy 16.57%
Sunitinib	VEGFR2	Thyroid dysfunction 0.00% to 84.80%, GI perforation 1.14% to 3.80%, pancreatitis 0.17% to 1.33%
Trametinib	MEK1/2	Retinopathy 0.00%
Trastuzumab	HER2	Peripheral neuropathy 19.20%
Trastuzumab emtansine	HER2 + Tubulin	ILD 4.02%, pancreatitis 0.45%
Vandetanib	VEGFR2	Thyroid dysfunction 49.40%, pancreatitis 0.43%
VEGFR	–	GI perforation 0.81%, pancreatitis 0.45%
VEGFRi monotherapy	–	Peripheral neuropathy 5.85%
Vemurafenib	BRAF V600E	ILD 7.53%, uveitis 0.29% to 5.00%, peripheral neuropathy 9.85%, retinopathy 0.42% to 2.85%
Vemurafenib + cobimetinib	BRAF V600E + MEK1/2	Uveitis 0.78%, retinopathy 10.61% to 25.51%

GI = Gastrointestinal, ILD = Interstitial lung disease.

While interpreting these research findings, it is essential to acknowledge the limitations of the available data. Given the relatively low incidence of autoimmune diseases, studies often involve small sample sizes, necessitating larger-scale research to validate these associations.

## 4. Potential mechanisms of TKI-induced autoimmune organ damage and systemic autoimmune diseases

While current investigations remain in their early stages, several key mechanisms have emerged, shedding light on the intricate relationship between TKIs and autoimmune diseases.

### 4.1. Imbalance in immune system regulation

TKIs may initiate autoimmune organ damage by disrupting the interactions among various cell populations within the immune system, leading to an imbalance in immune system regulation. Research suggests that certain TKIs may impact the function of T lymphocytes and B lymphocytes, inducing dysregulation in immune modulation and triggering inappropriate immune responses against normal tissues.^[6,54,55]^

### 4.2. Alterations in immune cell function

The direct impact of TKI class drugs may extend to various immune cells, including T lymphocytes, natural killer cells, and macrophages. These drugs may influence the functionality of these crucial immune cells,^[56,57]^ causing them to mount inappropriate aggressive responses against normal tissues and, consequently, leading to autoimmune organ damage.

### 4.3. Activation of inflammatory responses

TKIs may activate inflammatory responses, serving as a catalyst for autoimmune organ damage.^[58–60]^ Excessive activation of inflammatory responses can result in inflammation and damage to normal tissues, thereby initiating the development of autoimmune diseases. This mechanism may involve the release of inflammatory mediators and the activation of inflammatory pathways, creating a vicious cycle.

### 4.4. Exposure and attack of autoantigens

TKIs could interfere with the body’s recognition and tolerance mechanisms for self-antigens, causing the immune system to mistakenly identify normal tissues as foreign entities and mount an attack.^[61]^ The exposure and attack of autoantigens may lead to the occurrence of autoimmune organ damage through various pathways, including the activation of cytotoxic T lymphocytes and the production of autoantibodies.

### 4.5. Individual genetic susceptibility

Individual genetic susceptibility may play a pivotal role in TKI-induced autoimmune organ damage.^[62]^ Genetic variations resulting from individual differences in genetic backgrounds may render some individuals more prone to exaggerated immune responses to TKIs, thereby increasing the risk of autoimmune diseases. This explains why the same TKI treatment may induce autoimmune diseases in some patients while not triggering this adverse reaction in others.

The emergence and progression of systemic autoimmune diseases in the context of TKI therapy represent a complex interplay between the drug’s intended anticancer effects and unintended immune system repercussions. As these inhibitors modulate signaling pathways critical for cancer cell survival, they inadvertently impact immune cell behavior. The aberrant activation of immune cells can result in the initiation of autoimmune responses against multiple self-tissues throughout the body. This systemic involvement often manifests as a spectrum of autoimmune diseases, ranging from rheumatoid arthritis to systemic lupus erythematosus. Moreover, the disruption of immune tolerance may contribute to the systemic autoimmune effects of TKIs. Immune tolerance mechanisms, which normally prevent the immune system from attacking the body’s own tissues, can be compromised by these drugs.^[63]^ This breakdown in tolerance may allow autoreactive immune cells to escape regulation, leading to the widespread targeting of self-antigens and the initiation of autoimmune diseases affecting multiple organs. Additionally, the inflammatory responses triggered by TKIs can play a pivotal role in the development of systemic autoimmune diseases. The enhanced activity of immune cells and the release of pro-inflammatory mediators contribute to a systemic pro-inflammatory environment.^[64]^ This chronic inflammation can lead to the exacerbation of preautoimmune conditions or the de novo development of systemic autoimmune diseases, impacting various organs and tissues.

Understanding the factors influencing the development of systemic autoimmune diseases in the context of TKI therapy requires a comprehensive analysis of patient-specific characteristics, such as genetic predispositions and individual immune profiles. Furthermore, the type and duration of TKI treatment are crucial factors that may influence the likelihood and severity of systemic autoimmune manifestations.

The emergence and progression of systemic autoimmune diseases associated with TKI therapy result from the intricate interplay between the drug’s anticancer effects, immune system dysregulation, breakdown of tolerance mechanisms, and the pro-inflammatory environment. These potential mechanisms intertwine to form a complex network in the relationship between TKIs and autoimmune organ damage. Moreover, these mechanisms may manifest differently in various patient populations, influenced by individual biological differences and disease specificity.

## 5. Strategies for prevention and management of TKI-induced autoimmune diseases

The strategies aimed at preventing and managing TKI-induced autoimmune diseases seek to minimize adverse reactions while maintaining the efficacy of the drug in cancer treatment. While comprehensive preventive measures are still under development, several key strategies are gaining attention and undergoing active research.

### 5.1. Clinical monitoring and early intervention

Establishing regular clinical monitoring is crucial for early detection of TKI-induced autoimmune diseases. Periodic assessments of immune indicators, organ function, and clinical symptoms help identify potential issues promptly. Early intervention at the initial stages can effectively manage adverse reactions, alleviate symptoms, and prevent disease progression.^[65]^

### 5.2. Individualized treatment plans

Considering the impact of individual differences among patients, devising personalized treatment plans is particularly important.^[66]^ Based on a patient’s genetic characteristics, immune status, and other biological factors, physicians can adjust drug dosages or select specific TKIs to mitigate the risk of triggering autoimmune diseases.

### 5.3. Optimization and innovation of treatment strategies

Continuous optimization and innovation of treatment strategies are crucial in preventing TKI-induced autoimmune diseases. Improvements in drug formulations, exploration of combination therapies, and the development of novel treatment approaches contribute to a better balance between the anticancer effects of the drug and the stability of the immune system, thereby reducing potential adverse reactions.^[67]^

### 5.4. Patient education and communication

Patient education and communication are integral components of preventing and managing TKI-induced autoimmune diseases.^[68]^ Providing information to patients about potential adverse reactions, monitoring plans, and early symptoms enhances their understanding of the treatment process, empowering them to actively participate in clinical monitoring and management.

## 6. Future perspectives

For TKI-induced autoimmune diseases, future research directions need to span multiple dimensions to deepen our understanding of this complex phenomenon and enhance the precision and efficacy of treatment.

### 6.1. In-depth molecular mechanism studies

An in-depth exploration of how different TKIs affect the immune system’s regulation and function at the molecular level will be crucial for future research. By deciphering these molecular mechanisms, we can unveil the specific pathways through which TKIs trigger autoimmune diseases, providing a more precise foundation for targeted therapies.

### 6.2. Comprehensive integration of genetics and immunogenetics

Incorporating genetic and immunogenetic factors into clinical considerations and understanding the impact of individual differences on the immune effects of TKI can help develop more personalized treatment strategies, predict patient response to TKI, and identify potential adverse reactions in advance.

### 6.3. Development of novel TKIs

It also makes sense to develop novel TKIs that better balance their anticancer effects with immune system stability. The next generation of TKIs may possess increased selectivity, minimizing interference with normal immune function and consequently reducing the risk of autoimmune diseases.

### 6.4. Integration of immunotherapy

Exploring the possibility of combining immunotherapy with TKI treatment. Integrating immunotherapy may enhance immune system tolerance, mitigate the risk of autoimmune diseases, and simultaneously bolster the attack on cancer cells.

### 6.5. Advancement of large-scale clinical trials

Conducting larger-scale clinical trials to validate the effectiveness of different preventive and management strategies and gain a more comprehensive understanding of the incidence and manifestations of TKI-induced autoimmune diseases. This will provide a more robust foundation for developing feasible prevention and treatment approaches.

Through in-depth research in these directions, we aspire to comprehensively comprehend the mechanisms of TKI-induced autoimmune diseases, offering a more scientific basis for clinical practice. Ultimately, this will maximize the therapeutic efficacy of TKIs in cancer treatment while minimizing their potential adverse impacts.

In conclusion, despite the challenges posed by autoimmune diseases associated with TKIs, a combination of monitoring, personalized treatment, and innovative drug design strategies holds promise for effectively managing this issue. This comprehensive approach may be helpful to ensure minimal immune risks for patients while undergoing anticancer treatment.

## Acknowledgments

This study was funded by the Science and Technology Development Program of Jinan Municipal Health Commission (Grant number: 2023-Gong-32).

## Author contributions

**Conceptualization:** Xiao Li.

**Data curation:** Juan Shi, Zhenzhen Wei, Xiaolian Su.

**Formal analysis:** Juan Shi, Zhenzhen Wei.

**Funding acquisition:** Juan Shi.

**Investigation:** Juan Shi, Quancheng Ma, Zhenzhen Wei, Xiaolian Su.

**Supervision:** Qingyuan Fu, Xiao Li.

**Writing – original draft:** Juan Shi, Quancheng Ma.

**Writing – review & editing:** Qingyuan Fu, Xiao Li.
